# Dynamic trajectories in visual function and fall risk in older adults

**DOI:** 10.3389/fepid.2026.1910165

**Published:** 2026-07-27

**Authors:** Alan Y. Huang, Riaz Qureshi, Ali G. Hamedani

**Affiliations:** 1 Departments of Neurology, Ophthalmology, and Epidemiology, Perelman School of Medicine, University of Pennsylvania, Philadelphia, PA, United States; 2 Department of Ophthalmology, School of Medicine, University of Colorado Anschutz Medical Campus, Aurora, CO, United States; 3 Department of Epidemiology, School of Public Health, University of Colorado Anschutz Medical Campus, Aurora, CO, United States

**Keywords:** aging, contrast sensitivity, falls, visual acuity, visual impairment

## Abstract

**Purpose:**

Visual impairment is associated with falls in older adults, but whether changes in vision alter this risk is unclear.

**Methods:**

We performed a retrospective cohort study using data from the National Health and Aging Trends Study (NHATS), a nationally representative survey of Medicare beneficiaries aged ≥65 years. Distance acuity, near acuity, and contrast sensitivity were measured in 2022 and 2023. Change from 2022–2023 was calculated and categorized as stable (to ±0.1 logMAR), improving, or worsening. Using multivariable survey-weighted logistic regression, we compared the following fall-related outcomes in 2024: any fall in the last month, any fall in the last year, or multiple falls in the last year.

**Results:**

Among 6,327 eligible participants, distance acuity trends from 2022–2023 were stable in 50.3%, improved in 19.5%, and worsened in 30.2%. Similar patterns were observed for near acuity and contrast sensitivity. Worsening distance acuity was associated with higher odds of a fall in the past year (aOR 1.44, 95% CI 1.03–2.02), and worsening near acuity was associated with higher odds of a fall in the last month (aOR 1.44, 95% CI 1.3–2.02). Improvements in distance acuity, near acuity, and contrast sensitivity from 2022–23 were not associated with lower odds of falls in 2024.

**Conclusions:**

Worsening vision is associated with an increased risk of falls, but improving vision does not appear to reduce this risk. These findings suggest that while efforts to improve vision may not prevent falls, preventing a decline in visual function may help to reduce fall risk.

## 1 Introduction

Falls are the leading cause of injury-related morbidity and mortality among older adults in the United States. An estimated 14 million older adults fall each year, resulting in approximately 32,000 deaths, over 3 million emergency department visits, and healthcare costs exceeding $50 billion annually (1, 2). In addition to physical injury, falls precipitate downstream consequences ranging from fear of falling to loss of independence and functional decline (3). These downstream consequences can initiate a self-reinforcing cycle of activity restriction, deconditioning, and further falls, compounding the loss of independence and quality of life that often follows an initial fall.

Previous studies have identified visual impairment as a potential modifiable risk factor for falls in older adults due to the role of vision in postural control and navigation (4, 5). However, the majority of these studies have relied on a single, cross-sectional assessment of visual function (6–8). Vision changes over time, such as worsening as a result of age-related eye disease or improving via refraction and cataract surgery, but the extent to which these dynamic patterns affect fall risk has not been well studied (9). Understanding if worsening vision increases falls—and conversely, if vision improvement prevents falls—is important for developing fall prevention guidelines that effectively incorporate vision and eye health. To address these knowledge gaps, we used data from the National Health and Aging Trends Study (NHATS) to examine the association between longitudinal changes in distance acuity, near acuity, and contrast sensitivity and subsequent fall-related outcomes (10).

## 2 Methods

### 2.1 Study design

We analyzed data from the National Health and Aging Trends Study (NHATS), an ongoing, longitudinal, nationally representative study of Medicare beneficiaries aged 65 years and older (10, 11). A multistage probability sample of Medicare beneficiaries was enrolled in 2011, and participants are surveyed annually with replenishment of the sample in 2015, 2022, and 2023. For this analysis, we included participants who completed the round 12, 13, and 14 visits, corresponding to the years 2022–2024. Informed consent was previously obtained at the time of study enrollment and was not separately required for this secondary analysis. The study was adherent to the Declarations of Helsinki, and this study was exempt from additional institutional review board approval given its secondary analysis of de-identified, publicly available data.

### 2.2 Measurements

#### 2.2.1 Visual function assessment

Distance acuity, near acuity, and contrast sensitivity, were measured in NHATS using a tablet-based test with habitual correction (i.e., glasses or contact lenses). Distance and near acuity were recorded in logarithm of the minimum angle of resolution (logMAR) units, and contrast sensitivity in log contrast sensitivity (logCS) units, respectively. For each visual function measure, we subtracted the round 12 value from the round 13 value to calculate change in visual function for each subject. For distance and near acuity, a positive change (increase in logMAR) indicates worsening from round 12 to round 13; for contrast sensitivity, a negative change (decrease in logCS) indicates worsening. A 0.1 unit difference in logMAR is analogous to one line on a standard Snellen chart and was selected to represent a minimum clinically meaningful change (12). Therefore, we categorized change in logMAR as worsened if >0.1, improved if < −0.1, and stable if between −0.1 and 0.1. A similar 0.1 threshold definition was used for logCS.

#### 2.2.2 Outcome measures

Our primary outcomes at round 14 were: (1) any fall in the last month, (2) any fall in the past 12 months, and (3) multiple falls in the past 12 months. Secondary outcomes included fear of falling (worrying about falls in the last month) and activity limitation due to fear of falling (worrying about falls limited activities in the last month). All fall-related outcomes were self-reported via the NHATS surveys and answered as “Yes” or “No”. NHATS is administered in person by trained interviewers and permits a proxy respondent (typically a family member or caregiver) to answer on behalf of participants who are unable to respond reliably, including those with cognitive impairment (10). Analyses of each outcome were restricted to participants who did not have that outcome at round 12.

#### 2.2.3 Covariates

We adjusted for the following known risk factors for falls as potential confounders: age, gender, race/ethnicity, education, body mass index (BMI), hypertension, diabetes, heart attack or heart disease, stroke, lung disease, cancer, self-reported pain, and dementia. BMI was calculated from self-reported height and weight. Diagnoses of hypertension, diabetes, heart attack or heart disease, stroke, lung disease, and cancer were all self-reported in the NHATS surveys. Self-reported pain was classified as no pain, non-limiting pain, and limiting pain based on participant responses to questions asking if they have been bothered or limited by pain in the past month. To ascertain dementia, we used a previously validated algorithm that classifies dementia status as “probable dementia”, “possible dementia”, or “no dementia”; we defined participants as having dementia if they met criteria for “possible” or “probable dementia” (13).

### 2.3 Statistical analysis

**Figure 1:**
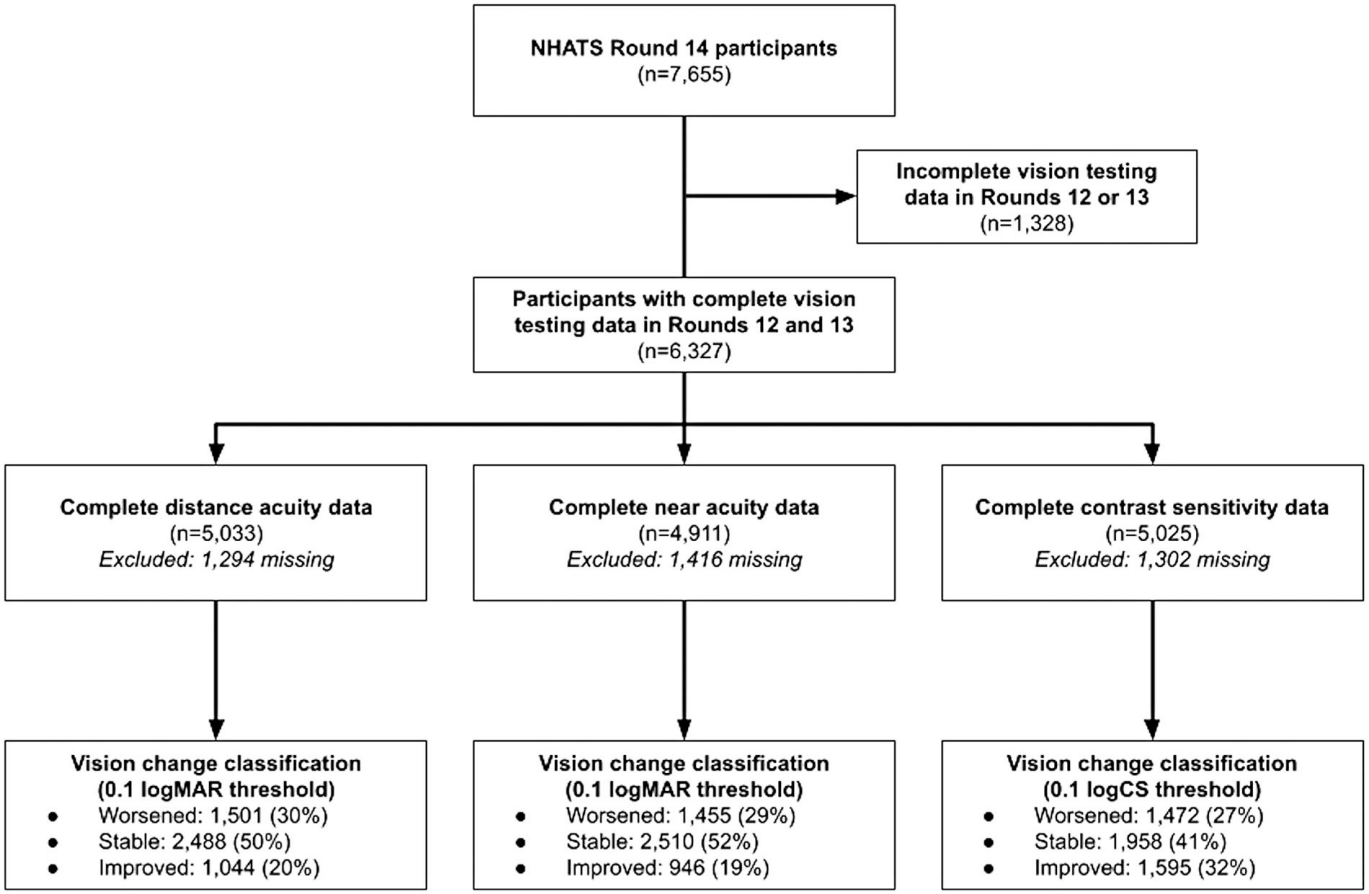
Flowchart of cohort selection from the National Health and Aging Trends Study 2022–2024.

Descriptive statistics were used to summarize baseline characteristics of NHATS participants with worsened, stable, or improved vision across all three metrics (distance acuity, near acuity, and contrast sensitivity). Logistic regression models were used to estimate unadjusted and adjusted odds ratios (ORs) with 95% confidence intervals and consisted of complete case analyses only. To account for the complex survey design of NHATS, all variance estimation was performed using modified balanced repeated replication (BRR) on sampling weights as recommended by NHATS (14, 15). Survey-weighted logistic regression model fit was confirmed using the built-in Stata link test for model specification. Imputation to account for missing data was not performed due to adequate sample size with complete case analyses. Of 7,655 round 14 participants, 1,328 (17.3%) were excluded for incomplete vision testing in round 12 or 13, yielding 6,327 eligible participants (Figure 1). All analyses were performed using StataNow SE (College Station, TX).

### 2.4 Sensitivity analyses

While a > 0.1-unit difference in logMAR is detectable in routine clinical practice, we considered the possibility that some changes of this magnitude could be due to test-retest variability rather than a true change in visual function, and clinically meaningful changes in logCS are not well defined. Therefore, we performed sensitivity analyses using >0.2 and >0.3 unit differences to define changes in visual function, with >0.3 being a common cutoff for clinical trial endpoints (16, 17). Additionally, because dementia could be considered a mediator rather than a covariate for the association between vision and falls, we performed a sensitivity analysis of all multivariate regression models excluding dementia as a covariate.

## 3 Results

For additional requirements for specific article types and further information please refer to “Article types” on every Frontiers journal page.

### 3.1 Baseline characteristics

A total of 6,327 NHATS participants met study inclusion criteria. A flowchart of cohort selection and missing data is depicted in Figure 1. Most participants were aged 65–74 years (weighted 46.3%). Women made up 55.1% of the cohort. By race and ethnicity, 78.5% of participants were White, 9.0% were Black, and 8.4% were Hispanic. Full baseline characteristics stratified by vision-change category are presented in Table 1. At the 0.1 logMAR/logCS threshold, distance acuity worsened in 30.2%, was stable in 50.3%, and improved in 19.5%. Similar patterns were observed for near acuity and contrast sensitivity.

**Table 1:** Baseline characteristics and prevalence of vision changes from Round 12 to 13 of NHATS (0.1 log unit threshold).

	Total population (*n* = 6,327)	Distance acuity change from R12-R13			Near acuity change from R12-R13			Contrast sensitivity change from R12–R13		
		Worsened (1,501; 30.2%)	No change (2,488; 50.3%)	Improved (1,044; 19.5%)	Worsened (1,455; 29.0%)	No change (2,510; 52.3%)	Improved (946; 18.7%)	Worsened (1,472; 27.0%)	No change (1,958; 40.6%)	Improved (1,595; 32.4%)
Age (*n*, weighted col%)										
65–69	308 (13.6%)	58 (13.3%)	160 (14.2%)	68 (13.5%)	61 (12.5%)	154 (14.1%)	65 (15.3%)	73 (10.9%)	129 (15.6%)	84 (14.1%)
70–74	749 (32.7%)	155 (30.0%)	393 (34.9%)	144 (30.9%)	131 (27.5%)	404 (35.0%)	139 (34.0%)	182 (28.1%)	333 (36.1%)	177 (33.1%)
75–79	1,149 (24.5%)	262 (27.3%)	576 (23.8%)	238 (25.1%)	248 (28.2%)	588 (23.9%)	218 (23.7%)	343 (28.9%)	443 (22.0%)	289 (25.1%)
80–84	1,171 (15.8%)	233 (14.9%)	602 (15.9%)	237 (16.7%)	237 (16.4%)	578 (15.3%)	225 (15.9%)	329 (17.0%)	453 (15.2%)	288 (15.6%)
85–89	774 (8.5%)	186 (9.5%)	346 (7.6%)	147 (8.4%)	171 (9.8%)	357 (7.9%)	133 (7.2%)	225 (9.5%)	275 (7.4%)	180 (8.2%)
90+	601 (4.9%)	132 (5.0%)	224 (3.6%)	117 (5.4%)	132 (5.6%)	242 (3.9%)	88 (3.9%)	170 (5.5%)	179 (3.7%)	123 (3.9%)
Female (*n*, weighted col%)	3,674 (55.1%)	862 (53.6%)	1,448 (56.0%)	580 (53.0%)	830 (53.7%)	1,426 (54.2%)	559 (57.3%)	842 (53.4%)	1,123 (55.8%)	919 (54.2%)
Race/ethnicity (*n*, weighted col%)										
White	4,025 (78.5%)	946 (78.3%)	1,713 (82.0%)	629 (75.6%)	924 (79.6%)	1,736 (82.2%)	578 (76.3%)	946 (78.9%)	1,359 (82.8%)	981 (76.4%)
Black	1,309 (9.0%)	311 (8.7%)	439 (7.4%)	230 (10.1%)	292 (8.1%)	451 (7.9%)	230 (10.5%)	284 (8.2%)	351 (7.9%)	343 (9.0%)
Other	196 (4.1%)	50 (4.1%)	63 (3.6%)	40 (5.2%)	50 (4.5%)	67 (3.5%)	30 (4.2%)	44 (3.5%)	52 (3.5%)	57 (5.1%)
Hispanic	729 (8.4%)	184 (8.9%)	249 (7.0%)	138 (9.1%)	165 (7.7%)	235 (6.3%)	104 (9.1%)	186 (9.4%)	180 (5.8%)	201 (9.4%)
Education (*n*, weighted col%)										
Less than high school	1,101 (12.1%)	276 (12.6%)	311 (8.2%)	197 (13.0%)	251 (12.7%)	318 (8.0%)	180 (13.1%)	255 (13.1%)	238 (7.3%)	286 (12.1%)
High school/some college	2,763 (43.5%)	651 (42.2%)	1,133 (44.1%)	455 (41.9%)	641 (42.5%)	1,112 (43.6%)	431 (41.8%)	645 (42.4%)	880 (42.8%)	712 (44.1%)
Bachelor/ associate	1,324 (26.0%)	330 (26.1%)	578 (27.3%)	239 (27.7%)	319 (26.2%)	598 (27.5%)	205 (27.3%)	334 (26.5%)	463 (27.8%)	350 (26.4%)
Master/ professional	945 (18.5%)	237 (19.0%)	450 (20.5%)	148 (17.4%)	224 (18.5%)	468 (20.8%)	127 (17.9%)	229 (18.0%)	365 (22.1%)	240 (17.4%)
BMI (*n*, weighted col%)										
<18.5	101 (2.1%)	22 (2.3%)	44 (2.0%)	21 (2.2%)	13 (1.4%)	50 (2.1%)	20 (3.1%)	22 (1.8%)	36 (2.2%)	29 (2.4%)
18.5–24.9	1,503 (31.9%)	344 (32.9%)	746 (32.0%)	299 (30.7%)	307 (31.4%)	771 (31.3%)	276 (33.8%)	432 (32.0%)	587 (31.9%)	370 (32.0%)
25–29.9	1,634 (36.3%)	346 (35.0%)	822 (37.0%)	330 (36.4%)	349 (39.5%)	812 (36.7%)	296 (32.3%)	461 (37.6%)	656 (37.5%)	379 (33.0%)
≥30.0	1,257 (29.7%)	277 (29.8%)	624 (28.9%)	265 (30.7%)	261 (27.7%)	632 (29.9%)	251 (30.8%)	359 (28.6%)	479 (28.3%)	328 (32.6%)
Hypertension (*n*, weighted col%)	3,498 (69.0%)	773 (70.0%)	1,680 (66.3%)	728 (71.4%)	754 (72.8%)	1,696 (67.4%)	651 (65.0%)	998 (70.7%)	1,325 (67.3%)	854 (66.9%)
Diabetes (*n*, weighted col%)	1,469 (27.4%)	332 (28.6%)	669 (25.1%)	322 (29.3%)	327 (27.8%)	694 (25.9%)	269 (28.3%)	427 (28.1%)	534 (25.5%)	362 (27.6%)
Heart attack/disease (*n*, weighted col%)	1,247 (22.1%)	283 (23.1%)	563 (19.6%)	263 (23.6%)	245 (20.6%)	605 (21.5%)	231 (21.3%)	345 (24.2%)	468 (19.7%)	295 (20.7%)
Stroke (*n*, weighted col%)	109 (2.2%)	33 (3.2%)	40 (1.6%)	21 (2.3%)	20 (2.9%)	61 (2.1%)	12 (1.5%)	44 (3.8%)	37 (1.9%)	13 (0.7%)
Lung disease (*n*, weighted col%)	1,096 (21.0%)	264 (22.3%)	503 (19.1%)	226 (23.2%)	240 (23.2%)	534 (19.6%)	189 (21.1%)	311 (19.1%)	415 (20.8%)	265 (22.1%)
Cancer (*n*, weighted col%)	349 (8.2%)	77 (9.0%)	178 (8.2%)	70 (8.0%)	87 (11.6%)	171 (7.7%)	60 (6.6%)	98 (9.1%)	149 (9.1%)	77 (6.2%)
Pain (*n*, weighted col%)										
No pain	1,909 (41.6%)	412 (41.7%)	951 (42.9%)	381 (38.7%)	421 (45.5%)	928 (40.8%)	347 (39.7%)	542 (41.3%)	747 (41.6%)	456 (42.5%)
Non-limiting pain	1,217 (25.7%)	277 (26.7%)	626 (25.9%)	239 (25.7%)	247 (26.2%)	645 (26.5%)	231 (25.7%)	324 (24.5%)	505 (28.0%)	312 (24.4%)
Limiting pain	1,519 (32.7%)	330 (31.7%)	718 (31.2%)	326 (35.6%)	305 (28.3%)	744 (32.7%)	285 (34.6%)	449 (34.2%)	552 (30.4%)	370 (33.0%)
Dementia (*n*, weighted col%)										
No dementia	4,662 (86.3%)	1,203 (87.2%)	2,188 (91.9%)	873 (89.0%)	1,160 (86.8%)	2,203 (92.2%)	803 (89.7%)	1,238 (89.3%)	1,716 (91.9%)	1,306 (88.1%)
Probable or possible	1,238 (13.7%)	298 (12.8%)	300 (8.1%)	171 (11.0%)	285 (13.2%)	307 (7.8%)	143 (10.3%)	234 (10.7%)	242 (8.1%)	289 (11.9%)

### 3.2 Association between vision changes and falls or fear of falling

Compared to those with stable vision, those whose near acuity worsened from round 12 to round 13 had an increased odds of falling in the last month (aOR 1.53, 95% CI 1.03–2.27) (Supplementary Table S1). Worsening distance acuity was associated with falls in the last year (aOR 1.44, 95% CI 1.3–2.02) but not in the last month (aOR 0.92, 95% CI 0.56–1.52). There was no association between worsening contrast sensitivity and fall outcomes. Likewise, improvement in distance acuity, near acuity, or contrast sensitivity was not associated with falls (Supplementary Table S1).

### 3.3 Sensitivity analyses

**Figure 2:**
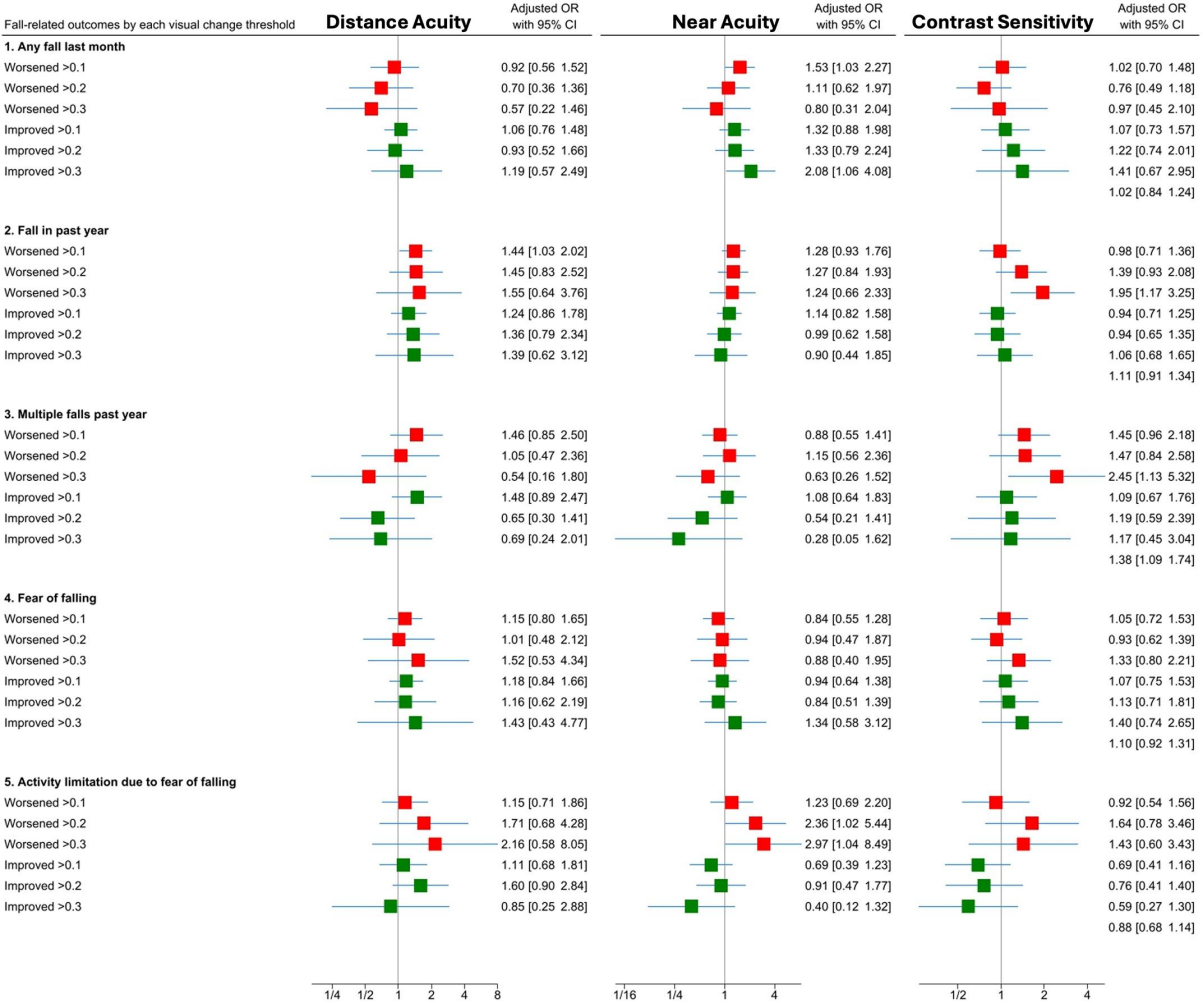
Sensitivity analysis of association between vision changes and fall-related outcomes at 0.1, 0.2, and 0.3 logMAR/logCS thresholds.

As expected, using logMAR/logCS thresholds of >0.2 or >0.3 resulted in a larger number of participants with stable vision (68.1–85.9%) and a smaller number with improved or worsened vision (Supplementary Tables S2–S3). Worsened near acuity was associated with activity limitation due to fear of falling in the last month at the >0.2 (aOR 2.36, 95% CI 1.02–5.42) and >0.3 (aOR 2.97, 95% CI 1.04–8.49) logMAR thresholds (Figure 2). A worsening of >0.3 in contrast sensitivity was significantly associated with any fall in the past year (aOR 1.95, 95% CI 1.17–3.26) and multiple falls in the past year (aOR 2.45, 95% CI 1.13–5.33). Paradoxically, improvement of >0.3 logMAR units in near acuity was associated with increased odds of falls in the last month (aOR 2.08, 95% CI 1.06–4.08). There were no other associations between changes in visual function across rounds 12–13 and fall outcomes in round 14. Sensitivity analyses of multivariate regressions excluding dementia as a covariate are shown in Supplementary Table S4 and showed no changes in outcomes compared to the multivariate regression with dementia as a covariate.

## 4 Discussion

In this nationally representative study of older U.S. adults, worsening visual function over a one-year interval was associated with increased fall risk, with a dose-response pattern across clinically meaningful visual change thresholds. Specifically, we found that worsened near and distance acuity of greater than 0.1 logMAR were associated with increased falls, and contrast sensitivity worsening of greater than 0.3 logMAR was associated with falls. However, improvement in vision did not protect against falls or other related outcomes. These findings suggest that temporal trajectories in visual function are uniquely associated with falls in older adults.

We found that worsening of distance acuity, near acuity, and contrast sensitivity from 2022–2023 were associated with fall-related outcomes in 2024. In the case of contrast sensitivity, there was the suggestion of a dose-response relationship between the amount of decline and reporting any fall in the past year: there was no association between >0.1 logCS decline and falls (aOR 0.98, 95% CI 0.71–1.36), but >0.2 logCS decline was associated with a higher point estimate (aOR 1.39, 95% CI 0.93–2.08) that increased and achieved statistical significance at the >0.3 logCS threshold (aOR 1.95, 95% CI 1.17–3.25). Previous cross-sectional studies have found that lower contrast sensitivity is associated with an increased risk of falls whereas distance and near visual acuity are not (6). This may be because contrast sensitivity is more important for detecting environmental hazards and modulating gait parameters such as stride length than visual acuity (18, 19). In a previous study using NHATS data, decline in near acuity between 2021–2022 was associated with greater fall risk in 2022, and decline in logCS between 2021–2022 was associated with an increased risk of multiple falls in 2022 (20). However, these findings were limited to a smaller cohort of people aged 70 and older with minimal temporal separation between the exposure (change in near acuity or logCS) and outcome (falls). A previous study of older community-residing women showed that declines in distance acuity was associated with greater odds of frequent falls in the subsequent year (21). Our study uses a similar framework to build on previous NHATS findings by separating the period in which changes in vision occurred (2022–2023) from the outcome ascertainment window (2024) and considering both magnitude and directionality of changes in vision. Together with a broader age range and larger sample size, this allowed us to reproduce known associations between change in logCS and falls with greater precision and uncover new relationships between declines in distance and near visual acuity and falls.

Because having lower levels of vision and experiencing a decline in visual function over time are both associated with an increased risk of falls, researchers have argued that interventions to improve vision (such as through refraction or cataract surgery) may lower the risk of falls. We did not find this to be the case: improvement in vision from 2022–2023 was not associated with better fall-related outcomes in 2024 regardless of how improvement was measured or defined, and improvement in near acuity >0.3 logMAR was associated with doubled adjusted odds of falls in the last month. Randomized controlled trials of vision-based fall prevention interventions are limited but have generally yielded null results, consistent with our findings (22). Proposed mechanisms behind this paradoxical relationship include sudden changes in visual input that cannot be adequately adapted to in older adults, as well as increased physical activity and exposure to hazards after vision improvement (5, 23). Additionally, previous studies have found large improvements in dioptric power and the use of multifocal spectacles to be associated with increased risk of falls, which may have contributed to our null results (19, 24, 25).

Several limitations merit consideration. First, falls were self-reported and therefore subject to recall bias and social desirability bias (i.e., falls could be perceived as a sign of frailty, which could lead to underreporting). We also used a complete case analysis; though the data were plausibly missing at random, we cannot exclude the possibility that participants with missing data differed from those analyzed. It is also important to consider possible residual confounding effects of dementia and cognitive decline given the associations with both sensory loss and falls; however, our sensitivity analysis without dementia as a covariate remained unchanged. The specific role of cognitive decline in our findings of vision loss and falls could be assessed with more granularity in future studies using individual cognitive measures found in NHATS as opposed to a dichotomous dementia covariate. Additionally, the one-year interval between changes in vision (2022–2023) and falls (2024) may not have been sufficient to capture all clinically meaningful effects. We were also unable to ascertain the reason for changes in vision (e.g., cataract surgery, disease progression, new refractive correction), and these could have heterogenous effects on fall risk. Nevertheless, the longitudinal and nationally representative nature of our study offers unique insights into how dynamic patterns of visual function may affect fall risk differently and suggests that interventions to prevent vision loss would have a greater impact on fall prevention than the correction of vision loss that has already occurred. From a public health standpoint, this could be implemented via routine eye examinations and early detection and treatment of progressive conditions such as glaucoma or diabetic retinopathy. Future studies should examine the efficacy of incorporating vision screening as a component of multifactorial fall-prevention programs and further explore the impact of specific causes of vision changes such as disease progression or cataract surgery on fall risk.

## Data Availability

The original contributions presented in the study are included in the article/Supplementary Material, further inquiries can be directed to the corresponding author.
