# Supplementary material for: Dynamic trajectories in visual function and fall risk in older adults
Source: Front Epidemiol. 2026 Jul 27;6:1910165. doi: 10.3389/fepid.2026.1910165 (PMC13454234; doi:10.3389/fepid.2026.1910165)
Supplement: Supplementary file 1 [file Table1.docx]

**Supplementary Table 1: Association between vision changes and fall-related outcomes in Round 14 for participants with no falls in R12 (0.1 log unit threshold)**

|  | Any fall in last month (R14) | | | >1 fall in past year (R14) | | | Multiple falls in past year (R14) | | | Fear of falling (R14) | | | Activity limitation due to fear of falling (R14) | | |
| --- | --- | --- | --- | --- | --- | --- | --- | --- | --- | --- | --- | --- | --- | --- | --- |
|  | Number of participants (N, %) | Unadjusted OR, 95% CI | Adjusted OR, 95% CI | Number of participants (N, %) | Unadjusted OR, 95% CI | Adjusted OR, 95% CI | Number of participants (N, %) | Unadjusted OR, 95% CI | Adjusted OR, 95% CI | Number of participants (N, %) | Unadjusted OR, 95% CI | Adjusted OR, 95% CI | Number of participants (N, %) | Unadjusted OR, 95% CI | Adjusted OR, 95% CI |
| Distance visual acuity |  |  |  |  |  |  |  |  |  |  |  |  |  |  |  |
| Worsened R12-13 | 103 (21.4%) | 0.98 (0.62-1.56) | 0.92 (0.56-1.52) | 183 (26.8%) | **1.50 (1.11-2.04)** | **1.44 (1.03-2.02)** | 123 (27.9%) | 1.40 (0.85-2.28) | 1.46 (0.85-2.49) | 130 (23.2%) | 1.23 (0.93-1.64) | 1.15 (0.80-1.64) | 82 (26.8%) | 1.16 (0.79-1.70) | 1.15 (0.71-1.86) |
| No change R12-13 | 204 (55.0%) | Ref | Ref | 359 (48.9%) | Ref | Ref | 227 (48.0%) | Ref | Ref | 316 (53.4%) | Ref | Ref | 168 (51.3%) | Ref | Ref |
| Improved R12-13 | 114 (23.6%) | 1.21 (0.89-1.65) | 1.06 (0.76-1.48) | 167 (24.3%) | 1.32 (0.97-1.79) | 1.24 (0.86-1.77) | 113 (24.1%) | 1.44 (0.91-2.27) | 1.48 (0.89-2.48) | 129 (23.3%) | 1.30 (0.90-1.88) | 1.18 (0.84-1.67) | 69 (22.0%) | 1.17 (0.73-1.85) | 1.11 (0.68-1.81) |
| Near visual acuity |  |  |  |  |  |  |  |  |  |  |  |  |  |  |  |
| Worsened R12-13 | 100 (27.5%) | **1.54 (1.05-2.27)** | **1.53 (1.03-2.27)** | 172 (24.7%) | 1.32 (0.99-1.76) | 1.28 (0.93-1.76) | 106 (21.5%) | 0.87 (0.55-1.35) | 0.88 (0.55-1.41) | 122 (19.4%) | 0.82 (0.57-1.17) | 0.84 (0.55-1.28) | 73 (27.1%) | 1.39 (0.81-2.41) | 1.23 (0.69-2.21) |
| No change R12-13 | 215 (50.2%) | Ref | Ref | 372 (55.2%) | Ref | Ref | 242 (55.3%) | Ref | Ref | 331 (61.1%) | Ref | Ref | 177 (56.7%) | Ref | Ref |
| Improved R12-13 | 93 (22.3%) | 1.28 (0.87-1.88) | 1.32 (0.88-1.98) | 144 (20.2%) | 1.12 (0.83-1.50) | 1.14 (0.82-1.58) | 100 (23.2%) | 1.05 (0.65-1.70) | 1.08 (0.64-1.84) | 108 (19.5%) | 0.89 (0.64-1.24) | 0.94 (0.64-1.38) | 53 (16.2%) | 0.86 (0.54-1.37) | 0.69 (0.39-1.24) |
| Contrast sensitivity |  |  |  |  |  |  |  |  |  |  |  |  |  |  |  |
| Worsened R12-13 | 130 (30.2%) | 1.12 (0.77-1.61) | 1.02 (0.70-1.48) | 227 (29.9%) | 1.02 (0.76-1.38) | 0.98 (0.70-1.35) | 156 (32.6%) | **1.45 (1.02-2.05)** | 1.45 (0.96-2.17) | 183 (31.0%) | 1.21 (0.87-1.69) | 1.05 (0.72-1.52) | 106 (30.8%) | 0.92 (0.55-1.54) | 0.92 (0.54-1.56) |
| No change R12-13 | 165 (43.3%) | Ref | Ref | 295 (45.1%) | Ref | Ref | 184 (43.2%) | Ref | Ref | 238 (42.9%) | Ref | Ref | 138 (46.0%) | Ref | Ref |
| Improved R12-13 | 124 (26.5%) | 1.10 (0.79-1.52) | 1.07 (0.73-1.57) | 188 (24.9%) | 0.92 (0.71-1.20) | 0.94 (0.71-1.26) | 121 (24.2%) | 1.13 (0.75-1.69) | 1.09 (0.67-1.75) | 154 (26.1%) | 1.13 (0.79-1.60) | 1.07 (0.75-1.54) | 75 (23.2%) | 0.82 (0.50-1.33) | 0.69 (0.41-1.16) |

Survey-weighted logistic regressions adjusted for age, gender, race/ethnicity, education, BMI, pain, dementia, and comorbidities (hypertension, diabetes, heart disease, stroke, lung disease, cancer).

**Supplementary Table 2: Baseline characteristics and prevalence of vision changes from Round 12 to 13 of NHATS (0.2 log unit threshold)**

|  | Total population (n= 6,327) | Distance acuity change from R12-R13 | | | Near acuity change from R12-R13 | | | Contrast sensitivity change from R12-R13 | | |
| --- | --- | --- | --- | --- | --- | --- | --- | --- | --- | --- |
|  |  | Worsened (768; 16.7%) | No change (3,900; 76.3%) | Improved (365; 6.9%) | Worsened (738; 16.0%) | No change (3,832; 77.9%) | Improved (331; 6.1%) | Worsened (672; 12.1%) | No change (3,438; 68.1%) | Improved (915; 19.8%) |
| **Age** (n, weighted col%) |  |  |  |  |  |  |  |  |  |  |
| 65-69 | 308 (13.6%) | 25 (15.5%) | 232 (13.6%) | 29 (14.5%) | 25 (16.2%) | 230 (13.4%) | 25 (19.0%) | 35 (13.7%) | 211 (13.9%) | 40 (13.6%) |
| 70-74 | 749 (32.7%) | 58 (30.0%) | 577 (32.9%) | 57 (36.5%) | 44 (24.3%) | 581 (33.8%) | 49 (35.3%) | 78 (29.0%) | 527 (33.0%) | 87 (36.9%) |
| 75-79 | 1,149 (24.5%) | 89 (28.2%) | 909 (25.0%) | 78 (20.1%) | 84 (28.8%) | 903 (25.0%) | 67 (17.7%) | 134 (23.6%) | 811 (25.3%) | 130 (23.0%) |
| 80-84 | 1,171 (15.8%) | 74 (13.5%) | 923 (16.2%) | 75 (14.4%) | 76 (14.9%) | 883 (15.7%) | 81 (15.7%) | 148 (16.6%) | 793 (15.9%) | 129 (14.6%) |
| 85-89 | 774 (8.5%) | 72 (8.2%) | 550 (8.1%) | 57 (9.6%) | 57 (9.1%) | 551 (8.0%) | 53 (8.5%) | 108 (10.9%) | 494 (7.9%) | 78 (7.6%) |
| 90+ | 601 (4.9%) | 50 (4.7%) | 386 (4.2%) | 37 (4.8%) | 54 (6.6%) | 378 (4.1%) | 30 (3.8%) | 84 (6.1%) | 328 (3.9%) | 60 (4.3%) |
| **Female** (n, weighted col%) | 3,674 (55.1%) | 439 (53.6%) | 2,261 (55.1%) | 190 (52.2%) | 422 (55.4%) | 2,194 (54.0%) | 199 (60.5%) | 372 (50.1%) | 1,990 (55.4%) | 522 (54.7%) |
| **Race/ethnicity** (n, weighted col%) |  |  |  |  |  |  |  |  |  |  |
| White | 4,025 (78.5%) | 461 (78.3%) | 2,634 (80.9%) | 193 (68.7%) | 441 (78.2%) | 2,612 (81.5%) | 185 (71.7%) | 426 (77.4%) | 2,327 (81.3%) | 533 (75.2%) |
| Black | 1,309 (9.0%) | 174 (8.8%) | 713 (7.7%) | 93 (14.0%) | 153 (7.2%) | 725 (8.4%) | 95 (12.3%) | 136 (8.8%) | 629 (7.9%) | 213 (9.4%) |
| Other | 196 (4.1%) | 24 (4.1%) | 109 (3.7%) | 20 (7.1%) | 30 (5.4%) | 107 (3.5%) | 10 (5.6%) | 14 (2.6%) | 100 (3.7%) | 39 (6.0%) |
| Hispanic | 729 (8.4%) | 104 (8.8%) | 412 (7.6%) | 55 (10.2%) | 105 (9.1%) | 360 (6.6%) | 39 (10.5%) | 92 (11.2%) | 355 (7.0%) | 120 (9.4%) |
| **Education** (n, weighted col%) |  |  |  |  |  |  |  |  |  |  |
| Less than high school | 1,101 (12.1%) | 155 (14.3%) | 545 (9.0%) | 84 (16.5%) | 147 (14.0%) | 524 (9.1%) | 78 (16.4%) | 137 (16.4%) | 454 (8.4%) | 188 (13.7%) |
| High school/some college | 2,763 (43.5%) | 340 (43.5%) | 1,754 (43.0%) | 145 (42.5%) | 332 (43.5%) | 1,701 (42.6%) | 151 (46.7%) | 284 (40.7%) | 1,561 (43.4%) | 392 (43.6%) |
| Bachelor/ associate | 1,324 (26.0%) | 171 (24.3%) | 893 (27.8%) | 83 (24.9%) | 155 (25.8%) | 900 (27.7%) | 67 (22.8%) | 154 (28.4%) | 788 (27.0%) | 205 (26.0%) |
| Master/ professional | 945 (18.5%) | 97 (17.9%) | 689 (20.1%) | 49 (16.1%) | 98 (16.7%) | 687 (20.6%) | 34 (14.1%) | 94 (14.6%) | 616 (21.1%) | 124 (16.7%) |
| **BMI** (n, weighted col%) |  |  |  |  |  |  |  |  |  |  |
| <18.5 | 101 (2.1%) | 7 (1.7%) | 69 (2.0%) | 11 (3.8%) | 6 (0.7%) | 66 (1.9%) | 11 (6.6%) | 11 (2.5%) | 64 (2.0%) | 12 (2.8%) |
| 18.5-24.9 | 1,503 (31.9%) | 116 (31.7%) | 1,168 (31.9%) | 105 (33.0%) | 104 (28.3%) | 1,140 (31.3%) | 110 (42.3%) | 204 (33.2%) | 1,018 (31.6%) | 167 (33.1%) |
| 25-29.9 | 1,634 (36.3%) | 121 (36.6%) | 1,258 (36.5%) | 119 (35.3%) | 121 (38.8%) | 1,250 (37.2%) | 86 (24.1%) | 190 (33.2%) | 1,133 (37.7%) | 173 (31.2%) |
| ≥30.0 | 1,257 (29.7%) | 107 (29.9%) | 974 (29.6%) | 85 (27.9%) | 88 (32.2%) | 970 (29.6%) | 86 (26.9%) | 158 (31.0%) | 855 (28.7%) | 153 (32.8%) |
| **Hypertension** (n, weighted col%) | 3,498 (69.0%) | 282 (73.6%) | 2,642 (67.5%) | 257 (70.3%) | 258 (73.3%) | 2,618 (68.2%) | 225 (61.2%) | 464 (76.6%) | 2,312 (66.8%) | 401 (67.8%) |
| **Diabetes** (n, weighted col%) | 1,469 (27.4%) | 134 (31.1%) | 1,074 (26.1%) | 115 (29.9%) | 126 (31.9%) | 1,056 (26.0%) | 108 (30.4%) | 207 (30.7%) | 951 (26.0%) | 165 (27.5%) |
| **Heart attack/disease** (n, weighted col%) | 1,247 (22.1%) | 98 (22.6%) | 924 (20.8%) | 87 (25.0%) | 80 (21.6%) | 923 (21.2%) | 78 (20.9%) | 162 (24.6%) | 807 (20.8%) | 139 (20.9%) |
| **Stroke** (n, weighted col%) | 109 (2.2%) | 14 (3.7%) | 71 (1.9%) | 9 (3.2%) | 9 (2.2%) | 79 (2.1%) | 5 (2.4%) | 19 (3.3%) | 69 (2.2%) | 6 (0.7%) |
| **Lung disease** (n, weighted col%) | 1,096 (21.0%) | 95 (21.0%) | 818 (20.2%) | 80 (26.1%) | 85 (27.8%) | 807 (19.1%) | 71 (32.8%) | 162 (21.4%) | 698 (20.3%) | 131 (22.0%) |
| **Cancer** (n, weighted col%) | 349 (8.2%) | 27 (11.6%) | 276 (8.1%) | 22 (8.2%) | 28 (11.0%) | 274 (8.4%) | 16 (4.0%) | 43 (8.6%) | 253 (9.1%) | 28 (3.7%) |
| **Pain** (n, weighted col%) |  |  |  |  |  |  |  |  |  |  |
| No pain | 1,909 (41.6%) | 145 (40.5%) | 1,473 (42.2%) | 126 (37.8%) | 154 (45.1%) | 1,423 (41.6%) | 119 (38.7%) | 230 (42.3%) | 1,308 (41.7%) | 207 (41.6%) |
| Non-limiting pain | 1,217 (25.7%) | 101 (23.4%) | 959 (26.5%) | 82 (24.3%) | 81 (27.0%) | 966 (26.5%) | 76 (22.0%) | 150 (24.3%) | 851 (26.5%) | 140 (25.1%) |
| Limiting pain | 1,519 (32.7%) | 119 (36.2%) | 1,131 (31.3%) | 124 (37.9%) | 102 (27.9%) | 1,125 (31.9%) | 107 (39.3%) | 202 (33.3%) | 994 (31.8%) | 175 (33.3%) |
| **Dementia** (n, weighted col%) |  |  |  |  |  |  |  |  |  |  |
| No dementia | 4,662 (86.3%) | 592 (86.4%) | 3,376 (91.0%) | 296 (86.6%) | 558 (85.6%) | 3,339 (91.6%) | 269 (83.9%) | 530 (85.4%) | 3,002 (91.5%) | 728 (87.6%) |
| Probable or  possible | 1,238 (13.7%) | 176 (13.6%) | 524 (9.0%) | 69 (13.4%) | 180 (14.4%) | 493 (8.4%) | 62 (16.1%) | 142 (14.6%) | 436 (8.5%) | 187 (12.4%) |

**Supplementary Table 3: Baseline characteristics and prevalence of vision changes from Round 12 to 13 of NHATS (0.3 log unit threshold)**

|  | Total population (n= 6,327) | Distance acuity change from R12-R13 | | | Near acuity change from R12-R13 | | | Contrast sensitivity change from R12-R13 | | |
| --- | --- | --- | --- | --- | --- | --- | --- | --- | --- | --- |
|  |  | Worsened (510; 11.7%) | No change (4,389; 85.9%) | Improved (134; 2.3%) | Worsened (501; 11.4%) | No change (4,261; 85.9%) | Improved (139; 2.7%) | Worsened (301; 5.8%) | No change (4,092; 79.5%) | Improved (632; 14.7%) |
| **Age** (n, weighted col%) |  |  |  |  |  |  |  |  |  |  |
| 65-69 | 308 (13.6%) | 9 (13.6%) | 261 (13.7%) | 16 (19.5%) | 9 (12.2%) | 260 (14.0%) | 11 (17.2%) | 18 (15.9%) | 246 (13.8%) | 22 (12.7%) |
| 70-74 | 749 (32.7%) | 20 (30.3%) | 657 (33.2%) | 15 (27.9%) | 18 (24.2%) | 635 (33.1%) | 21 (42.5%) | 34 (27.8%) | 614 (33.1%) | 44 (36.4%) |
| 75-79 | 1,149 (24.5%) | 30 (29.3%) | 1,019 (24.9%) | 27 (19.0%) | 26 (27.0%) | 1,003 (25.0%) | 25 (14.2%) | 67 (28.1%) | 945 (24.6%) | 63 (24.6%) |
| 80-84 | 1,171 (15.8%) | 26 (11.7%) | 1,022 (15.9%) | 24 (17.0%) | 31 (16.0%) | 980 (15.7%) | 29 (12.4%) | 60 (14.7%) | 942 (16.0%) | 68 (15.0%) |
| 85-89 | 774 (8.5%) | 31 (9.5%) | 628 (8.1%) | 20 (10.0%) | 29 (13.2%) | 608 (8.0%) | 24 (8.9%) | 40 (8.6%) | 603 (8.3%) | 37 (6.8%) |
| 90+ | 601 (4.9%) | 21 (5.6%) | 434 (4.2%) | 18 (6.6%) | 23 (7.3%) | 423 (4.1%) | 16 (4.9%) | 32 (4.9%) | 413 (4.2%) | 27 (4.5%) |
| **Female** (n, weighted col%) | 3,674 (55.1%) | 288 (54.4%) | 2,537 (54.9%) | 65 (45.5%) | 291 (58.2%) | 2,438 (53.8%) | 86 (67.8%) | 162 (47.1%) | 2,364 (54.9%) | 358 (56.1%) |
| **Race/ethnicity** (n, weighted col%) |  |  |  |  |  |  |  |  |  |  |
| White | 4,025 (78.5%) | 302 (77.6%) | 2,920 (80.2%) | 66 (67.5%) | 293 (78.7%) | 2,865 (80.9%) | 80 (69.3%) | 185 (78.1%) | 2,722 (80.3%) | 379 (76.7%) |
| Black | 1,309 (9.0%) | 110 (8.7%) | 831 (8.1%) | 39 (16.4%) | 110 (8.1%) | 828 (8.4%) | 35 (10.4%) | 61 (8.3%) | 786 (8.5%) | 131 (7.6%) |
| Other | 196 (4.1%) | 19 (4.8%) | 127 (3.9%) | 7 (6.6%) | 20 (5.0%) | 121 (3.6%) | 6 (11.0%) | 6 (2.0%) | 119 (3.8%) | 28 (6.1%) |
| Hispanic | 729 (8.4%) | 75 (8.9%) | 476 (7.8%) | 20 (9.6%) | 73 (8.2%) | 415 (7.0%) | 16 (9.4%) | 47 (11.7%) | 431 (7.4%) | 89 (9.7%) |
| **Education** (n, weighted col%) |  |  |  |  |  |  |  |  |  |  |
| Less than high school | 1,101 (12.1%) | 112 (14.8%) | 638 (9.6%) | 34 (18.5%) | 107 (14.5%) | 605 (9.5%) | 37 (19.4%) | 61 (14.8%) | 586 (9.4%) | 132 (14.0%) |
| High school/some college | 2,763 (43.5%) | 225 (43.9%) | 1,961 (42.8%) | 53 (48.8%) | 227 (44.4%) | 1,896 (42.9%) | 61 (39.9%) | 127 (43.3%) | 1,827 (42.6%) | 283 (46.1%) |
| Bachelor/ associate | 1,324 (26.0%) | 106 (24.0%) | 1,011 (27.6%) | 30 (20.7%) | 101 (23.3%) | 993 (27.6%) | 28 (27.6%) | 74 (29.5%) | 937 (27.5%) | 136 (23.5%) |
| Master/ professional | 945 (18.5%) | 63 (17.4%) | 757 (19.9%) | 15 (11.9%) | 63 (17.9%) | 744 (20.0%) | 12 (13.2%) | 37 (12.4%) | 720 (20.5%) | 77 (16.4%) |
| **BMI** (n, weighted col%) |  |  |  |  |  |  |  |  |  |  |
| <18.5 | 101 (2.1%) | 3 (0.8%) | 80 (2.1%) | 4 (5.7%) | 2 (0.2%) | 73 (1.9%) | 8 (10.6%) | 3 (1.4%) | 76 (2.1%) | 8 (2.6%) |
| 18.5-24.9 | 1,503 (31.9%) | 44 (27.9%) | 1,310 (32.3%) | 35 (25.2%) | 36 (21.8%) | 1,271 (31.5%) | 47 (51.8%) | 94 (35.7%) | 1,218 (31.8%) | 77 (30.4%) |
| 25-29.9 | 1,634 (36.3%) | 40 (34.0%) | 1,422 (36.7%) | 36 (29.9%) | 46 (42.8%) | 1,383 (37.0%) | 28 (12.3%) | 76 (28.9%) | 1,324 (36.8%) | 96 (36.8%) |
| ≥30.0 | 1,257 (29.7%) | 41 (37.3%) | 1,086 (29.0%) | 39 (39.2%) | 37 (35.3%) | 1,070 (29.6%) | 37 (25.3%) | 70 (34.0%) | 1,021 (29.2%) | 75 (30.2%) |
| **Hypertension** (n, weighted col%) | 3,498 (69.0%) | 109 (80.5%) | 2,975 (67.6%) | 97 (75.7%) | 102 (75.5%) | 2,911 (68.3%) | 88 (56.3%) | 198 (77.6%) | 2,778 (67.4%) | 201 (70.4%) |
| **Diabetes** (n, weighted col%) | 1,469 (27.4%) | 58 (36.5%) | 1,230 (26.3%) | 35 (32.3%) | 56 (38.2%) | 1,187 (26.2%) | 47 (35.4%) | 90 (32.0%) | 1,147 (26.2%) | 86 (30.3%) |
| **Heart attack/disease** (n, weighted col%) | 1,247 (22.1%) | 41 (26.2%) | 1,034 (20.7%) | 34 (36.7%) | 26 (18.9%) | 1,023 (21.4%) | 32 (17.8%) | 78 (26.3%) | 968 (21.2%) | 62 (18.1%) |
| **Stroke** (n, weighted col%) | 109 (2.2%) | 6 (5.2%) | 87 (2.1%) | 1 (0.4%) | 2 (0.4%) | 87 (2.1%) | 4 (4.7%) | 10 (4.4%) | 83 (2.1%) | 1 (0.1%) |
| **Lung disease** (n, weighted col%) | 1,096 (21.0%) | 42 (22.7%) | 925 (20.6%) | 26 (21.0%) | 35 (25.4%) | 902 (20.0%) | 26 (38.0%) | 64 (19.9%) | 862 (20.6%) | 65 (21.3%) |
| **Cancer** (n, weighted col%) | 349 (8.2%) | 11 (10.4%) | 308 (8.4%) | 6 (4.9%) | 9 (10.6%) | 302 (8.4%) | 7 (4.2%) | 14 (8.4%) | 298 (8.8%) | 12 (2.3%) |
| **Pain** (n, weighted col%) |  |  |  |  |  |  |  |  |  |  |
| No pain | 1,909 (41.6%) | 50 (34.2%) | 1,651 (42.0%) | 43 (38.8%) | 57 (40.1%) | 1,587 (41.6%) | 52 (44.3%) | 100 (40.9%) | 1,537 (41.4%) | 108 (46.8%) |
| Non-limiting pain | 1,217 (25.7%) | 37 (32.0%) | 1,075 (26.1%) | 30 (17.3%) | 34 (27.8%) | 1,060 (26.5%) | 29 (18.8%) | 52 (22.4%) | 1,019 (26.6%) | 70 (21.9%) |
| Limiting pain | 1,519 (32.7%) | 50 (33.8%) | 1,277 (31.9%) | 47 (43.9%) | 43 (32.1%) | 1,248 (31.9%) | 43 (36.9%) | 98 (36.7%) | 1,191 (32.0%) | 82 (31.2%) |
| **Dementia** (n, weighted col%) |  |  |  |  |  |  |  |  |  |  |
| No dementia | 4,662 (86.3%) | 375 (85.2%) | 3,779 (90.6%) | 110 (88.7%) | 370 (84.8%) | 3,688 (91.2%) | 108 (80.0%) | 236 (82.7%) | 3,531 (90.8%) | 493 (88.2%) |
| Probable or  possible | 1,238 (13.7%) | 135 (14.8%) | 610 (9.4%) | 24 (11.3%) | 131 (15.2%) | 573 (8.8%) | 31 (20.0%) | 65 (17.3%) | 561 (9.2%) | 139 (11.8%) |

### **Supplementary Table 4: Sensitivity analysis of associations between vision changes and fall-related outcomes in Round 14 with no dementia covariate (0.1 log unit threshold)**

|  | Any fall in last month (R14) | | | >1 fall in past year (R14) | | | Multiple falls in past year (R14) | | | Fear of falling (R14) | | | Activity limitation due to fear of falling (R14) | | |
| --- | --- | --- | --- | --- | --- | --- | --- | --- | --- | --- | --- | --- | --- | --- | --- |
|  | Number of participants (N, %) | Unadjusted OR, 95% CI | Adjusted OR, 95% CI | Number of participants (N, %) | Unadjusted OR, 95% CI | Adjusted OR, 95% CI | Number of participants (N, %) | Unadjusted OR, 95% CI | Adjusted OR, 95% CI | Number of participants (N, %) | Unadjusted OR, 95% CI | Adjusted OR, 95% CI | Number of participants (N, %) | Unadjusted OR, 95% CI | Adjusted OR, 95% CI |
| Distance visual acuity |  |  |  |  |  |  |  |  |  |  |  |  |  |  |  |
| Worsened R12-13 | 103 (21.4%) | 0.98 (0.62-1.56) | 0.92 (0.56-1.52) | 183 (26.8%) | **1.50 (1.11-2.04)** | **1.47 (1.05-2.06)** | 123 (27.9%) | 1.40 (0.85-2.28) | 1.46 (0.85-2.51) | 130 (23.2%) | 1.23 (0.93-1.64) | 1.15 (0.81-1.65) | 82 (26.8%) | 1.16 (0.79-1.70) | 1.16 (0.72-1.88) |
| No change R12-13 | 204 (55.0%) | Ref | Ref | 359 (48.9%) | Ref | Ref | 227 (48.0%) | Ref | Ref | 316 (53.4%) | Ref | Ref | 168 (51.3%) | Ref | Ref |
| Improved R12-13 | 114 (23.6%) | 1.21 (0.89-1.65) | 1.06 (0.75-1.48) | 167 (24.3%) | 1.32 (0.97-1.79) | 1.24 (0.87-1.78) | 113 (24.1%) | 1.44 (0.91-2.27) | 1.49 (0.89-2.49) | 129 (23.3%) | 1.30 (0.90-1.88) | 1.19 (0.84-1.68) | 69 (22.0%) | 1.17 (0.73-1.85) | 1.11 (0.69-1.78) |
| Near visual acuity |  |  |  |  |  |  |  |  |  |  |  |  |  |  |  |
| Worsened R12-13 | 100 (27.5%) | **1.54 (1.05-2.27)** | **1.52 (1.03-2.25)** | 172 (24.7%) | 1.32 (0.99-1.76) | 1.32 (0.97-1.80) | 106 (21.5%) | 0.87 (0.55-1.35) | 0.90 (0.57-1.43) | 122 (19.4%) | 0.82 (0.57-1.17) | 0.85 (0.56-1.29) | 73 (27.1%) | 1.39 (0.81-2.41) | 1.24 (0.69-2.24) |
| No change R12-13 | 215 (50.2%) | Ref | Ref | 372 (55.2%) | Ref | Ref | 242 (55.3%) | Ref | Ref | 331 (61.1%) | Ref | Ref | 177 (56.7%) | Ref | Ref |
| Improved R12-13 | 93 (22.3%) | 1.28 (0.87-1.88) | 1.32 (0.88-1.97) | 144 (20.2%) | 1.12 (0.83-1.50) | 1.14 (0.82-1.58) | 100 (23.2%) | 1.05 (0.65-1.70) | 1.09 (0.64-1.84) | 108 (19.5%) | 0.89 (0.64-1.24) | 0.94 (0.64-1.38) | 53 (16.2%) | 0.86 (0.54-1.37) | 0.70 (0.39-1.24) |
| Contrast sensitivity |  |  |  |  |  |  |  |  |  |  |  |  |  |  |  |
| Worsened R12-13 | 130 (30.2%) | 1.12 (0.77-1.61) | 1.02 (0.70-1.48) | 227 (29.9%) | 1.02 (0.76-1.38) | 0.98 (0.71-1.36) | 156 (32.6%) | **1.45 (1.02-2.05)** | 1.44 (0.96-2.18) | 183 (31.0%) | 1.21 (0.87-1.69) | 1.05 (0.72-1.53) | 106 (30.8%) | 0.92 (0.55-1.54) | 0.93 (0.55-1.57) |
| No change R12-13 | 165 (43.3%) | Ref | Ref | 295 (45.1%) | Ref | Ref | 184 (43.2%) | Ref | Ref | 238 (42.9%) | Ref | Ref | 138 (46.0%) | Ref | Ref |
| Improved R12-13 | 124 (26.5%) | 1.10 (0.79-1.52) | 1.07 (0.73-1.56) | 188 (24.9%) | 0.92 (0.71-1.20) | 0.95 (0.72-1.26) | 121 (24.2%) | 1.13 (0.75-1.69) | 1.09 (0.68-1.75) | 154 (26.1%) | 1.13 (0.79-1.60) | 1.08 (0.76-1.54) | 75 (23.2%) | 0.82 (0.50-1.33) | 0.69 (0.41-1.17) |

Survey-weighted logistic regressions adjusted for age, gender, race/ethnicity, education, BMI, pain, and comorbidities (hypertension, diabetes, heart disease, stroke, lung disease, cancer). Dementia excluded as covariate per sensitivity analysis.
